# Can human cardiovascular regulation during exercise be learnt from feedback from arterial baroreceptors?

**DOI:** 10.1113/expphysiol.2007.037879

**Published:** 2007-04-20

**Authors:** Mari Herigstad, George M Balanos, Peter A Robbins

**Affiliations:** Department of Physiology, Anatomy and Genetics, University of Oxford UK

## Abstract

During dynamic exercise, a large fall in systemic vascular resistance occurs. Arterial pressure (AP) is, however, maintained through a combination of central command and neural activity from muscle afferents that adjust the autonomic outflow to the circulation. How these signals are calibrated to provide accurate regulation of AP remains unclear. This study tests the hypothesis that the calibration can be ‘learnt’ through feedback from the arterial baroreceptors arising over multiple trials of exercise. Eight healthy subjects undertook three different protocols in random order. The test protocol consisted of 7 days' training, when subjects were exposed on 70 occasions to 4 min of exercise (50% of maximal oxygen uptake capacity) paired with neck suction (−40 mmHg) to mimic an excessive rise in AP at the carotid baroreceptors with exercise. Two control protocols involved training with either exercise or neck suction alone. No significant changes in mean AP, diastolic AP or heart rate during normal exercise were detected following training with any protocol. However, the rise in systolic AP with exercise was attenuated by an average of 7.3 ± 2.0 mmHg (mean ± s.e.m., *P* < 0.01) on the first and second days following training with the test protocol, but not with either control protocol (*P* < 0.05 for difference between protocols, ANOVA). In conclusion, this study failed to show that mean AP during normal exercise could be reduced through prior conditioning by overstimulation of the baroreceptors during exercise. However, a reduction in systolic AP was observed that suggests the presence of some plasticity within the autonomic response, consistent with our hypothesis.

During dynamic muscular exercise, there is a large fall in peripheral vascular resistance to accommodate the increase in blood flow that is necessary to meet the increase in metabolic demand. Despite this fall in peripheral vascular resistance which, on its own, would be expected to cause a fall in mean arterial pressure (MAP; Ardell *et al.* 1980; Strange *et al.* 1990; Thomas & Segal, 2004), MAP either remains constant or increases slightly (DiCarlo & Bishop, 1990; Buckwalter & Clifford, 1999; Querry *et al.* 2001*a*). The maintenance of arterial pressure arises through a combination of central command (Goodwin *et al.* 1972; Schibye *et al.* 1981; Gallagher *et al.* 2001) and afferent activity from working muscle (the afferent arm of the pressor reflex; Alam & Smirk, 1937; Coote *et al.* 1971; McCloskey & Mitchell, 1972; Mitchell *et al.* 1983), which increases sympathetic tone (and reduces parasympathetic tone) in relation to exercise intensity.

Both central command and afferents from the working muscle may be viewed as feedforward signals for the control of blood pressure, in the sense that alterations of blood pressure do not immediately and directly alter the signals themselves. Indeed, feedback of MAP cannot explain the maintenance of blood pressure during exercise because there is no fall in MAP to act as an error signal to drive the system. Nevertheless, experimentally it is clear that the arterial baroreceptors play an important role in maintaining arterial pressure because a large fall in arterial pressure occurs with exercise in experimental animals whose arterial baroreceptors have been denervated (Ardell *et al.* 1980; Melcher & Donald, 1981; DiCarlo & Bishop, 1992), although the data in relation to human studies are less clear cut (Smit *et al.* 2002; Joyner, 2006). In order to explain this observation, it has been proposed that the feedforward signals (central command and afferent activity from working muscles) exert their effects by resetting the arterial baroreflex so that a given blood pressure is associated with a higher level of sympathetic tone to the cardiovascular system. There is now a considerable body of evidence to support this hypothesis (Ebert, 1986; Potts *et al.* 1993; Papelier *et al.* 1994; Gallagher *et al.* 2001; Raven *et al.* 2006).

Over recent years, a considerable body of research has developed surrounding the central neural substrate for the interactions between central command, the exercise pressor reflex and the baroreflex. A particular focus of this has been on the role of GABAergic inhibitory mechanisms within the nucleus tractus solitarius in resetting the arterial baroreflex (Potts, 2006). However, while resetting of the arterial baroreflex by central command and afferent nerve activity from working muscle provides an attractive hypothesis to explain many of the experimental findings, there remains a difficulty in the sense that there is no explanation of how these feedforward signals are accurately calibrated to provide a resetting of the arterial baroreflex that is appropriate. Too little resetting would result in a lower blood pressure with exercise, and too much would result in an excessive rise in blood pressure with exercise. More specifically, if GABAergic inhibitory processes do indeed underlie resetting of the arterial baroreflex with exercise, this observation still provides no explanation of how the appropriate synaptic weightings develop in order to produce the desired physiological outcome. The present study sought to test the hypothesis that the calibration of the feedforward signals is learnt through repetitive trials of exercise, with feedback on performance arising from the arterial baroreceptors. In a sense, this hypothesis is no different from our current understanding of how accurate control of other motor tasks is achieved, i.e. through a combination of practice and sensory feedback. The novelty of the hypothesis in this particular setting is that the sense organs are the arterial baroreceptors and the motor output is autonomic in nature.

In order to test our hypothesis, subjects undertook a period of training in which repeated bouts of exercise were paired with altered sensory feedback from the arterial baroreceptors that was generated by using neck suction (Ernsting & Parry, 1957). Blood pressure and heart rate responses to a normal period of exercise were measured before and after this period of training in order to determine whether they had been modified. The responses were compared with those to two control protocols, one involving repeated periods of exercise only, and the other involving repeated periods of altered sensory feedback from the baroreceptors under conditions of rest as opposed to exercise.

## Methods

Eight healthy male subjects (18–30 years of age) completed this study (Table 1). All were non-smoking, none was taking any regular medication and none had a history of cardiovascular or respiratory disease. All took some degree of regular exercise, but none was particularly athletic or highly trained. The study conformed to the provisions of the Declaration of Helsinki and was approved by the Oxfordshire Clinical Research Ethics Committee.

**Table 1:** Characteristics of volunteers

Subject no.	Age (years)	Height (cm)	Weight (kg)	*V̇*_O_2_max_ (l min^−1^)	*V̇*_O_2_max_ (ml kg^−1^ min^−1^)	Workload (W)	HR (beats min^−1^)	ΔSBP (mmHg)	ΔDBP (mmHg)	ΔHR (beats min^−1^)
1	26	174	77	3.3	42.9	120	134.4	−7.0	−10.8	−3.3
2	26	188	80	3.0	37.5	120	120.1	−9.6	−6.4	4.0
3	21	180	83	2.7	32.5	80	116.8	−8.4	−7.4	0.6
4	26	183	78	3.0	38.5	80	118.9	−11.1	−6.5	−6.7
5	27	182	67	3.2	47.8	100	111.0	−9.4	−5.4	−1.1
6	25	183	90	3.2	35.6	100	111.0	−5.0	−14.2	−3.6
7	27	177	64	3.9	60.9	120	141.2	−5.3	−10.7	−3.3
8	23	183	80	2.8	35.0	80	124.7	−17.9	−6.6	4.1
Average	25.1	181.3	77.4	3.1	41.3	100.0	122.3	−9.2	−8.5	−1.2
s.d.	2.1	4.3	8.4	0.4	9.3	18.5	10.8	4.1	3.1	3.8

*V̇*_0_2_max_, maximal oxygen uptake capacity; HR, heart rate; ΔSBP, change in systolic blood pressure; ΔDBP, change in diastolic blood pressure; and ΔHR, change in heart rate. Workload is the level of exercise chosen to correspond to 50% of the individual maximal oxygen uptake capacity values. Values for ΔSBP, ΔDBP and ΔHR are those associated with −40 mmHg of neck suction applied at rest and were determined from the preliminary study. Values for HR are averages during exercise from pretraining days −2 and −1, and post-training days +1 and +2 for protocol EX.

### Protocols

Each subject undertook a preliminary study and, if found suitable, they then undertook the three main protocols of the study. The order of the three main protocols was randomized between the subjects. There are six possible orders in which three protocols can be undertaken. The first six subjects each undertook one of these orders at random. The final two subjects undertook the protocols in the orders that were drawn for the first two subjects. There was always an interval of at least 2 weeks between subjects undertaking any two protocols.

In the preliminary study, subjects were familiarized with the procedure for generating neck suction to stimulate the carotid baroreceptors. Owing to variations in anatomy, including variations in tissue composition and thickness, and the location of the carotid bifurcation (Querry *et al.* 2001*b*), not all the potential subjects showed a good response to the neck suction. The response was assessed using a 4 min exposure to neck suction of −40 mmHg and averaging systolic blood pressure (SBP) over the last 3 min. In general, subjects either responded not at all to the neck suction or with a drop in SBP of > 5 mmHg. For this reason, a fall in SBP of ≥ 5 mmHg was used as an inclusion criterion for the study. Changes in heart rate (HR) were not employed as an inclusion/exclusion criterion because the response of HR to neck suction is generally considered to be transient (Ogoh *et al.* 2003*b*). In this study, roughly one in three potential subjects did not drop their SBP sufficiently to be included in the study. Following this familiarization period, subjects then undertook an incremental exercise test to exhaustion on a cycle ergometer during which breath-to-breath oxygen consumption was recorded (Pandit & Robbins, 1992). This test was used to determine the level of exercise to be employed (50% of maximal oxygen uptake capacity) in the three main protocols of the study.

**Figure 1:**
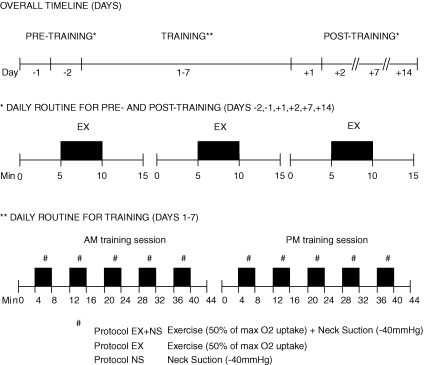
Overall scheme for each of the three protocols of the study Protocols differ only in the design of the daily routine for training. The pretraining measurements and post-training measurements were common to all protocols.

The protocols differ from each other only in relation to the 7 day training regime (Fig. 1). In EX+NS, exercise (50% of maximal oxygen uptake capacity on a cycle ergometer) was paired with neck suction (−40 mmHg). In EX, exercise was undertaken in the training periods without any associated neck suction. In NS, neck suction was undertaken in the training periods without any associated exercise. Subjects came to the laboratory twice a day to undertake the training periods associated with each protocol. On each day, the two training periods were separated from each other by at least 4 h. The times were the same for each day during the entire training regime for each subject, although these differed somewhat between subjects. Each training session consisted of five repetitions of 4 min bouts of exercise paired with neck suction (EX+NS) or exercise only (EX) or neck suction only (NS). Each repetition was separated from the next by 4 min of rest. Outside the laboratory, subjects were asked to refrain from anything but very mild exercise, and were asked to wear a heart rate monitor which would warn them if their heart rate rose above 100 beats min^−1^.

Measurements of the cardiovascular response to exercise were made before and after the training regimes to determine whether the training regime had altered the response. Pretraining measurements were undertaken on the 2 days before training (days −2 and −1), and the post-training measurements on the first and second days after training (days +1 and +2), and 1 week (day +7) and 2 weeks (day +14) after training. The procedures followed were identical on each of these days for all protocols. Three 15 min measurement periods were employed, with the subject undertaking exercise at 50% of maximal oxygen uptake capacity during minutes 5–10 of each period. During these measurement periods, the subjects' blood pressure (using an automated upper-arm sphygmomanometer) and heart rate (using a 3-lead ECG) were measured.

### Apparatus and techniques

All exercise was undertaken on a modified electrically braked cycle ergometer (Mijnhardt KEM3, Cardiokinetics, Salford, UK). Rather than sit on a saddle, subjects sat on a narrow ‘bench press’ seat with a backrest to undertake the exercise. This position was more convenient for undertaking neck suction during exercise and also allowed the subjects' arms to be positioned so that their forearms rested on arm supports with the upper arms vertical. This enabled measurements of arterial pressure using automated sphygmomanometers to be undertaken in a repeatable and reliable manner. The sphygmomanometers used were an Omron 705CP (Henfield, UK) for the first two subjects and an Omron M5-1 (Henfield, UK) for the remaining six subjects.

Neck suction was applied through a lead cuff strapped to the anterior part of the neck. The neck cuff was connected to a 25 l reservoir in which a stable negative pressure could be maintained. The negative pressure in the reservoir was generated by a vacuum cleaner connected to the reservoir via a laboratory-built Starling resistor. The negative pressure in the volume surrounding the Starling resistor was maintained constant using a feedback control mechanism comprised of a pressure sensor which could open or close a solenoid valve linked to the suction from the vacuum cleaner. The pressure in the reservoir was checked using a manometer to ensure that it remained at −40 mmHg, as set via the negative pressure surrounding the Starling resistor.

### Data analysis

For each variable of interest (SBP, diastolic blood pressure (DBP), MAP and HR), the response to exercise for each measurement period was calculated as the difference between the last 3 min of the exercise period and the last 3 min of the period of rest immediately before the exercise period. These responses were then averaged across measurement periods, as appropriate, before further analysis. The significance of any differences between the cardiovascular responses to exercise before and after training between protocols was assessed by using analysis of variance (ANOVA). An initial ANOVA was conducted on the data from each protocol separately to determine whether the training period had any effect on the responses observed. A further ANOVA was conducted on the pooled data across protocols to determine whether any effects of training differed between protocols. In this ANOVA, the fixed factors were those of protocol and training (before *versus* after), with subjects as a random factor. Whether training had an effect that differed between protocols was assessed from the significance of the interactive term between factors of protocol and training and, in particular, the contrast between EX+NS and the two control protocols (EX and NS). Statistical significance was assumed at *P* < 0.05 for this study.

## Results

Of the eight subjects originally recruited to the main study, all managed to complete the control measurements (day −1 and day −2), the training periods and the immediate post-training measurements (day +1 and day +2) for all three protocols. However, three subjects missed one or more follow-up measurements at day +7 and day +14. The shortest period within which any subject completed all three protocols was 3 months. The longest period was 1 year. Table 1 shows the maximal oxygen consumption for the subjects as measured in the preliminary experiments, and the work rate associated with half-maximal oxygen consumption at which the exercise components of this study were conducted. Table 1 also shows their SBP, DBP and HR responses to −40 mmHg of neck suction under conditions of rest.

### Training

**Figure 2:**
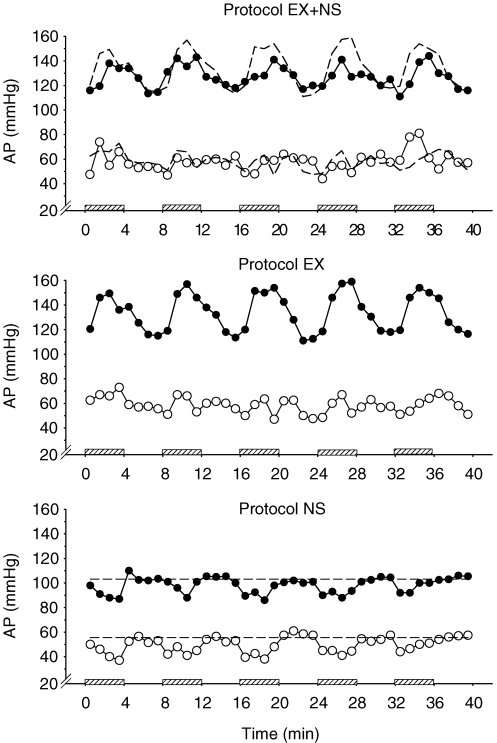
Changes in systolic pressure (SBP) and diastolic pressure (DBP) during an example training session from each protocol for one subject The hatched bars indicate 4 min periods of stimulus (neck suction and exercise for EX+NS; exercise only for EX; neck suction only for NS), with 4 min recovery periods in between. Dashed lines, shown for EX+NS and NS, illustrate the expected SBP and DBP when the effect of neck suction is removed. For EX+NS, the dashed lines represent the values taken from the corresponding EX training session. For NS, the dashed lines represent mean values for SBP and DBP, calculated by averaging the values for the last 3 min of the recovery periods during the training session.

Figure 2 shows an example of the variations that occurred in SBP and DBP during 1 day of training for one subject for each of the three protocols. The reduction in SBP and DBP with neck suction can be seen in the data from NS. The increment in SBP with exercise can be seen in EX. A comparison of the responses to EX+NS with those to EX shows that lower values for SBP were obtained when neck suction was applied concomitantly with the exercise stimulus.

### Pre- and post-training measurements

**Figure 3:**
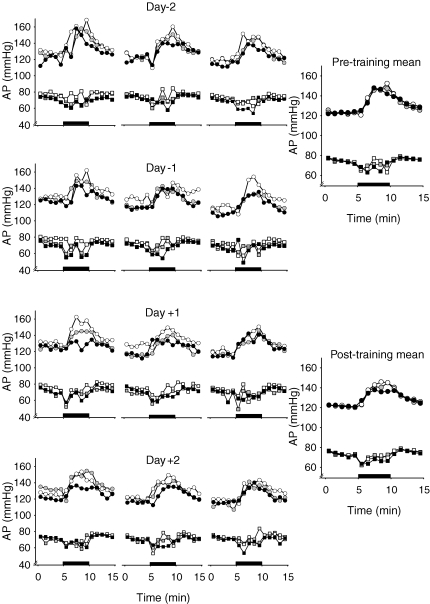
Response of systolic pressure (SBP) and diastolic pressure (DBP) to exercise before (days −2 and −1) and after training (days +1 and +2) for each of the three protocols for one subject Filled symbols, EX+NS; shaded symbols, EX; open symbols, NS. Circles, SBP; squares, DBP. Filled bars indicate 5 min periods of exercise. Three plots for each day show the three repeats of the measurement period. Also shown are the average values pretraining and post-training. [These averages have been slightly adjusted so that the average resting blood pressure (minutes 1–5) is exactly the same for each protocol. This aids comparison of the changes in blood pressure with exercise between protocols.]

Figure 3 shows, for one subject, the SBP and DBP responses to exercise before (days −2 and −1) and after (days +1 and +2) training for each of the three protocols. It illustrates the substantial variability in both the SBP and DBP responses, which was a general feature observed in all subjects. However, the averaged values also suggest that, overall, the increment in SBP with exercise may be a little less for EX+NS compared with the other two protocols.

**Figure 4:**
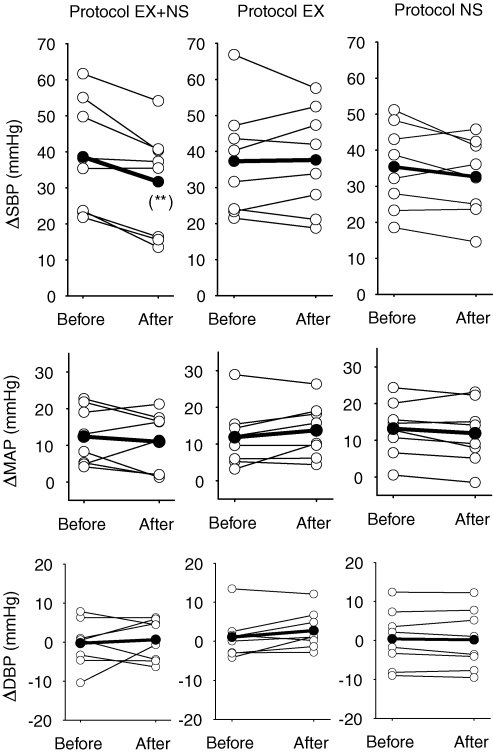
Changes in systolic pressure (ΔSBP), mean blood pressure (ΔMAP) and diastolic pressure (ΔDBP) on going from rest to exercise before and after training for each protocol Open symbols, results for individual subjects (data for before training, average of all values for days −2 and −1; data for after training, average of all values for days +1 and +2); filled symbols, mean values across subjects. ** ΔSBP for EX+NS lower after training compared with before training (*P* < 0.01), and different from changes in ΔSBP with training in other protocols (*P* < 0.05).

**Figure 5:**
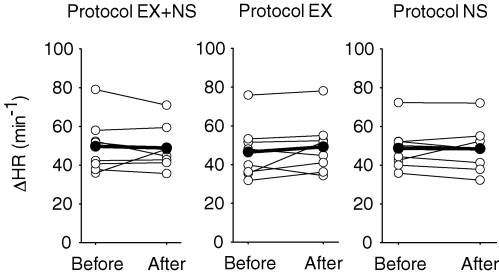
Changes in heart rate (ΔHR) on going from rest to exercise before and after training for each protocol Open symbols, results for individual subjects (data for before training, average of all values for days −2 and −1; data for after training, average of all values for days +1 and +2); filled symbols, mean values across subjects. No significant differences in ΔHR were detected between protocols.

The average SBP for the last 3 min of exercise was calculated and subtracted from the average SBP for the last 3 min of rest immediately preceding the exercise period. This gave the increment in SBP (ΔSBP) with exercise. An analogous procedure was adopted for DBP, to give the increment in DBP (ΔDBP) with exercise. The MAP was calculated as one-third of SBP plus two-thirds of DBP. The above calculations were repeated for MAP to determine the increment in MAP (ΔMAP) with exercise. For each protocol and variable, the six values for the rest-to-exercise transitions for days −2 and −1 were averaged to give an average control value for each protocol, and the six values for the rest-to-exercise transitions for days +1 and +2 were averaged to give an average value for the post-training period (Fig. 4). Similar calculations were performed for HR (Fig. 5).

For EX+NS, six out of the eight subjects showed a clear fall in ΔSBP, one showed a very small fall, and one showed a very small rise. The average fall in ΔSBP was 7.3 ± 2.0 mmHg (mean ± s.e.m., *P* < 0.01). This overall fall in ΔSBP following training with EX+NS did not differ between day +1 (7.2 ± 1.9 mmHg, *P* < 0.01) and day +2 (7.5 ± 2.3 mmHg, *P* < 0.02). For EX, four out of the eight subjects showed a fall in ΔSBP, and the other four a rise. The average fall in ΔSBP was −0.8 ± 2.1 mmHg, which was not significant. For NS, five of the eight subjects showed a fall in ΔSBP, and the three others a rise. The average fall in ΔSBP was 3.7 ± 1.8 mmHg, which was not significant. ANOVA demonstrated that the response of ΔSBP to EX+NS was different from the responses to EX and NS (*P* < 0.05).

Follow-up measurements, after the subjects had resumed their normal pattern of exercise activity, were obtained on day +7 and day +14 in most subjects. On both days there were no significant differences in ΔSBP compared with control pretraining measurements (i.e. days −2 and −1) for any protocol (falls in ΔSBP were −0.2 ± 0.7, −2.0 ± 4.8 and 1.2 ± 1.4 mmHg for EX+NS, EX and NS, respectively).

For the changes in DBP (ΔDBP, Fig. 4), MAP (ΔMAP, Fig. 4) and HR (ΔHR, Fig. 5), the effects following training with all protocols were small and inconsistent. No significant differences in these variables were detected for any protocol.

## Discussion

The results of this study have not provided a completely clear outcome in relation to our hypothesis. On the one hand, following training with EX+NS there was no alteration in the rise in MAP with exercise. Thus our null hypothesis, that training with EX+NS would have no effect on MAP, should not be rejected. On the other hand, training with EX+NS did reduce the normal increment in SBP that occurs with exercise. This effect was not reproduced by control training periods in which either neck suction or exercise were employed as single stimuli. This suggests that the normal pressor response to exercise can nevertheless be modified by a training period in which the normal baroreceptor feedback present during exercise has been modified. We conclude that our findings do suggest some element of ‘plasticity’ in the pressor response to exercise based on feedback arising from the arterial baroreceptors, but our findings fall short of demonstrating that an appropriate pressor response to exercise can be ‘learnt’ through the feedback that arises from the arterial baroreceptors during the repeated trials of exercise that occur as a normal part of daily life.

### Limitations of end-point of study

The main end-points of this study were measurements of SBP and DBP during rest and exercise. One difficulty associated with these as end-points is that there is no single ‘systolic’ or ‘diastolic’ arterial pressure within the arterial compartment of the cardiovascular system (O'Rourke, 1990). Substantial differences in pressure arise between central and peripheral arteries because of factors such as arterial compliance and resistance, coupled with the effects of pressure wave reflection. In their review of blood pressure measurement during exercise, Griffin *et al.* (1997) recommended that direct measurements of ascending aorta pressures should ideally be used as a standard. However, each of the three protocols required the measurement of arterial pressure on six different days (days −2, −1, +1, +2, +7 and +14), and so each subject would have had to have undergone 18 separate catheterizations to make these measurements directly. This was not considered ethically acceptable in normal subjects undertaking a physiological study. However, when direct measurements of ascending aortic pressure are not possible, Griffin and co-workers recommend that either manual or automated sphygmomanometry should be used for the assessment of SBP during exercise. For our purposes, the automated cuff has the advantage over traditional auscultation because it removes any opportunity for observer bias in relation to the three protocols. We did, however, periodically check that there were no marked differences between traditional auscultation and the automatic cuff.

Although satisfactory for measurements of SBP, Griffin *et al.* (1997) consider that cuff-based measurements of DBP during exercise underestimate DBP in the ascending aorta. Thus our values for DBP and MAP are likely to be, at least to some degree, an underestimate of the value pertaining to the ascending aorta. To counter this problem, an important aspect of our experimental design is that each subject acts as their own control. With such a design, the important outcome measure is not the absolute value for DBP, but rather the change in DBP following an intervention. Thus the requirement for our measurement of DBP is not one of absolute accuracy, but rather that any over- or underestimation of DBP is consistent within a given subject. Even so, it is perhaps of note that, for the subject depicted in Fig. 2, neck suction had clear effects on both SBP and DBP at rest, but only on SBP during exercise. Thus one possible reason for failing to detect changes in DBP during exercise following EX+NS might simply be inadequacy within the method for assessing changes in DBP.

A further limitation of the study is that a reduction in SBP following training with EX+NS can potentially be explained by mechanisms other than recalibration of the autonomic response to exercise within the CNS. For example, it is possible that EX+NS simply alters baroreflex function, while training with either EX or NS on its own does not. In more general terms, our experimental design was directed towards determining whether or not a particular phenomenon relating to integrative cardiovascular control could be detected. The experimental design is not capable of providing very much by way of insight into the underlying mechanisms, for which an altogether more reductive approach would be required.

### Limitations of training procedures

If the more general physiological hypothesis of this study is correct, then the calibration of the resetting of the arterial baroreflex in response to signals arising through central command and afferent activity from working muscle is likely to have begun in infancy and continued throughout growth and development and into adulthood. From this perspective, our training period of 7 days appears very short. As a consequence, it is important to recognize that the absence of an effect of EX+NS on the increment in MAP with exercise post-training in our study should not be taken as evidence that such an effect would still be absent over substantially longer time scales.

In practice, the length of the training period was dictated by what we felt our subjects might reasonably tolerate. Each day of training not only required twice daily attendance at the laboratory, but also that the subjects voluntarily restricted themselves to undertaking only the lightest of exercise outside the laboratory. Without this restriction, any effect associated with the training periods could be reversed by normal activity. Given the relative brevity of the training procedure, it would seem reasonable to view the reduction in the increment in SBP with exercise after EX+NS as signifying some degree of response, and not the total response that might occur were it possible to extend the training over a much longer period of time.

Another complication of training is that neck suction only really alters the perception of blood pressure at the carotid baroreceptors, and not at the aortic baroreceptors (Davos *et al.* 2002). Thus the afferent information regarding blood pressure during exercise has only been partly altered in the present study, and a more satisfactory experimental design might be one in which it is possible to alter all afferent activity that is sensitive to arterial pressure simultaneously. However, studies on animals suggest that the carotid baroreflex effects may dominate in relation to a number of key target sites for vascular regulation (Oberg & White, 1970; Pelletier *et al.* 1971). Also, we observed that the reduction in blood pressure, although not necessarily HR, with neck suction was maintained over the 4 min training period. Similar observations have been made in several other studies using the technique of neck suction, suggesting that there was relatively little or no ‘buffering’ effect from the aortic baroreceptors (Ernsting & Parry, 1957; Bevegard & Shepherd, 1966).

### Absence of effects on heart rate

In the case of HR, the changes that are predicted by our hypothesis are less easy to formulate. First, less resetting of the baroreflex during exercise might reasonably result in a smaller rise in HR with exercise. However, any reduction in blood pressure associated with a reduction in resetting of the baroreflex would serve to offset this effect. Second, the effect of neck suction on HR was not consistent across all our subjects (Table 1). This observation is in accord with other studies that have found that neck suction had a more marked effect on blood pressure than on HR (Bevegard & Shepherd, 1966; Mancia *et al.* 1977), and also that any bradycardia induced by neck suction may be transitory (Ernsting & Parry, 1957; Mancia *et al.* 1977; Ogoh *et al.* 2003*a*). One explanation of these latter findings (but see also Fadel *et al.* 2003) is that the aortic baroreceptors may play a more significant role in the regulation of HR (Mancia *et al.* 1977; Ferguson *et al.* 1985) as distinct from some of the other influences on arterial pressure, in particular vascular tone. If so, then the conditioning stimulus of neck suction is likely to be less successful in resetting the heart rate response to exercise compared with resetting the overall blood pressure response to exercise.

### Effects of physical training

Both the intensity and the duration of the exercise training regime in the present study were chosen so as to have little or no effect on physical fitness. However, more intense training over longer time periods can alter physical fitness and this is associated with alterations in the cardiovascular response to exercise (Davies *et al.* 1970). Of particular note, Klausen *et al.* (1982) found that, following 8 weeks of single-leg training of both legs, HR was reduced considerably more during submaximal work with either leg singly than during submaximal work with both legs together. They concluded that, since the muscles involved in the two types of exercise probably are identical and had been identically trained, then this would indicate that the training effect on HR response to steady-state dynamic exercise depends on a central mechanism. While their experiment is somewhat removed from the present one (which, in addition, found no effect on HR), it nevertheless indicates that the HR response can be changed through a period of training in a way that is quite specific to a particular motor command (one-legged *versus* two-legged exercise), the specificity suggesting CNS involvement.

The findings of Klausen *et al.* (1982) also raise an important question in relation to our present hypothesis, and that is to what degree learning a cardiovascular response to a particular type or intensity of exercise may influence the cardiovascular response to another type or intensity of exercise. We cannot know the answer to this question, but, by analogy with the learning of other motor tasks, the answer is most likely to depend on the degree of similarity between the two tasks. If the patterns of motor activity are very similar, then learning an appropriate sympathetic output to the cardiovascular system for one task is likely to aid the accomplishment of this for the other task. However, if the patterns of motor activity differ substantially between the two tasks, then this is less likely to be the case.

### Comparison with studies of respiratory control during exercise

During mild-to-moderate exercise in humans, pulmonary ventilation is precisely matched to metabolism so that arterial values for partial pressures of CO_2_ and O_2_ remain remarkably constant. In 1992, Somjen proposed that the feedforward mechanisms that increase ventilation during exercise were effectively a learned response, with feedback on performance arising from the chemoreceptors (Somjen, 1992). This hypothesis has many similarities to that of the present study, although in Somjen's case the output is neural discharge to the respiratory muscles rather than autonomic tone, and the sense organs which enable learning to occur are the chemoreceptors rather than the baroreceptors. Experimental support for Somjen's hypothesis has come from experiments in both animals (Martin & Mitchell, 1993) and man (Wood *et al.* 2003). Conceptually, the design of the present study is very similar to the design employed by those studies, but in the present case the stimulus paired with exercise is an increase in carotid baroreceptor activity rather than an increase in chemoreceptor activity.

### Clinical implications

If the hypothesis underlying this study is correct, it has important implications for understanding certain pathophysiological responses to cardiovascular disease. For example, after a myocardial infarction that resulted in a degree of post-ischaemic heart failure, it is likely that the neural processes underlying baroreceptor resetting, if unaltered, would not generate the same changes in arterial pressure as before. Consequently, as a patient takes exercise in the period after the development of heart failure, a gradual modulation of baroreceptor resetting with exercise would be expected to occur. We know of no study that has directly looked for such changes in baroreceptor resetting in this situation. However, a number of studies have described a progressive change in baroreflex function at rest over a period of exercise training after myocardial infarction (La Rovere *et al.* 1992, 2002; Mimura *et al.* 2005). Furthermore, an increase in baroreflex sensitivity with exercise training post-infarction is associated with an improved prognosis (La Rovere *et al.* 2002). Coupling these results with those of the present study suggests that trying to understand changes in exercise-induced baroreceptor resetting after the onset of heart failure might be a fruitful area to explore.

### Concluding remarks

This study sought to explore the hypothesis that an appropriate autonomic response is a learned component of the cardiovascular response to exercise, with arterial baroreceptors acting as the sense organ that enables feedback on performance to be monitored. The study was not successful in demonstrating that prior conditioning with EX+NS brings about a reduction in the increase in MAP that occurs with exercise. However, the study did suggest that conditioning with EX+NS, as distinct from conditioning with either EX or NS separately, reduces the increment in SBP with exercise, a change that would be consistent with our hypothesis. Clearly, further studies would be required to determine whether our hypothesis is correct. Most likely, such studies would need to find a way to extend and improve the period of conditioning with the paired stimuli of exercise and altered ‘perception’ of arterial pressure.
